# Influence of Bath Hydrodynamics on the Micromechanical Properties of Electrodeposited Nickel-Cobalt Alloys

**DOI:** 10.3390/ma14143898

**Published:** 2021-07-13

**Authors:** Isman Khazi, Ulrich Mescheder, Jürgen Wilde

**Affiliations:** 1 Institute for Microsystems Technology (iMST), Faculty of Mechanical & Medical Engineering, Robert Gerwig-Platz 1, 78120 Furtwangen im Schwarzwald, Germany; mes@hs-furtwangen.de; 2 Department of Microsystems Engineering (IMTEK), University of Freiburg, Georges-Köhler-Allee 103, 79110 Freiburg im Breisgau, Germany; juergen.wilde@imtek.uni-freiburg.de; 3 Associated to the Faculty of Engineering, University of Freiburg, 79110 Freiburg im Breisgau, Germany

**Keywords:** nanocrystalline nickel-cobalt alloys, electrochemical deposition, bath hydrodynamics, diffusion layer thickness, anomalous co-deposition, chronopotentiometry, nanocrystalline alloys, grain size, microhardness, Hall–Petch relationship

## Abstract

The influence of bath hydrodynamics on the resultant micromechanical properties of electrodeposited nickel-cobalt alloy system is investigated. The bath hydrodynamics realized by magnetic stirring is simulated using COMSOL Multiphysics and a region of minimum variation in velocity within the electrolytic cell is determined and validated experimentally. Nickel-cobalt alloy and nickel coating samples are deposited galvanostatically (50 mA/cm^2^) with varying bath velocity (0 to 42 cm/s). The surface morphology of samples gradually changed from granular (fractal dimension 2.97) to more planar (fractal dimension 2.15) growth type, and the according average roughness decreased from 207.5 nm to 11 nm on increasing the electrolyte velocity from 0 to 42 cm/s for nickel-cobalt alloys; a similar trend was also found in the case of nickel coatings. The calculated grain size from the X-ray diffractograms decreased from 31 nm to 12 nm and from 69 nm to 26 nm as function of increasing velocity (up to 42 cm/s) for nickel-cobalt and nickel coatings, respectively. Consecutively, the measured Vickers microhardness values increased by 76% (i.e., from 393 HV0.01 to 692 HV0.01) and by 49% (i.e., from 255 HV0.01 to 381 HV0.01) for nickel-cobalt and nickel coatings, respectively, which fits well with the Hall–Petch relation.

## 1. Introduction

The use of electrochemical deposition (ECD) technique in combination with silicon microtechnology opened new realms of applications by expanding the choice of materials for microfabrication by integrating the use of metals, alloys, and composites [1,2,3,4]. Using the simple UV-LIGA method, the generation of high aspect ratio microstructures (HARMS) is made possible for, e.g., fabrication of microtools such as micropunches, electrochemical machining (ECM) microtools, micromolds, etc. [5,6,7]. Moreover, using ECD, functional coatings can also be deposited, such as magnetic coatings and hard and wear-resistant protective coatings, etc. [1,2,3,5,6,7,8,9,10,11]. Furthermore, as described by Eliaz and Glieadi [12] the status of ECD has been elevated from mere art to more empirical technology, which is exemplified with the use of ECD for depositing copper interconnects in microprocessors. Apart from the wide choice of materials which can be electrodeposited, the ECD technique itself is quite simple when compared to other deposition techniques used for the fabrication of MEMS, such as physical and chemical vapor deposition, as it requires a relatively simple, low-cost setup, with deposition being carried out in an electrolyte (liquid) at an atmospheric pressure and near ambient temperatures [12]. Although nickel electrodeposition has been extensively used for the microfabrication (since the inception of the LIGA technique [1]) of mechanically robust microstructures; however, the micromechanical properties of electrodeposited Ni (eNi) are not sufficient for specific applications, where high microhardness, etc., is required for, e.g., in the case of microtools [13]. Therefore, owing to their excellent micromechanical, tribological, and magnetic properties, the electrochemically deposited nickel-cobalt (eNiCo) alloys have gathered special attention of the research community in the recent decades as a promising candidate for the generation of microstructures for micromechanical applications. This is further evident by series of review papers from the year 2019 by authors of this paper [14] and Karimzadeh et al. [9], in the year 2020 by Mbugua et al. [15] and Safavi et al. [16], and very recently in 2021 by Omar et al. [17], all of which comprehensively described the current and prospective applications of eNiCo alloys.

The majority of research groups have attributed the excellent micromechanical properties of eNiCo alloys, additionally to their alloy composition, i.e., the solid solution strengthening effect, to their nanocrystalline (nC) microstructure, wherein the materials with grain size < 100 nm are categorized as nC-materials [18,19,20]. For, e.g., comparing two eNiCo alloys with similar alloy composition, the alloy with finer grain size possesses higher microhardness values [14]; i.e., complying with the Hall–Petch relation [21]. Here, the higher grain boundary area hinders the dislocation movement to a higher extent when compared to the coarse-grained materials, thereby enhancing the micromechanical and tribological properties [18,22]. It is generally accepted that the use of ECD technique can be exploited for the fabrication of nC metals, alloys, and composites by the engineering of the grain size at the atomistic level by carefully controlling and adjusting the ECD operating conditions, such as the applied current density, grain refiners, pulse plating method, etc. [23,24,25], where the primary requirement for the fabrication of nC-materials is the massive nucleation rate (resulting in nuclei with confined grain size), rather than the nucleation growth. However, the grain size engineering becomes complex for ECD of alloys and composite materials, due to presence of more than one active species in the electrolyte [26].

The influence of some ECD conditions, such as electrolyte metal content, applied current density, bath additives as grain refiners, pulse plating, and pulse reverse plating on the resultant grain size for the fabrication of nC-eNiCo alloys is summarized in our previous review paper [14]. From the comprehensive review, it was found that the electrolyte cobalt content (Co_E_), organic bath additives, applied current density, pulse plating deposition mode, and the electrolyte flow rate tend to have an influence on the grain size (i.e., they influence the nucleation and growth kinetics). However, a model-based correlation of the ECD operating conditions with the resultant microstructural properties was missing. Additionally, contradictory results among the research findings were found, which could be attributed to the intricate correlation of the eNiCo alloy system with the ECD operating conditions [14]. Additionally, the influence of bath hydrodynamics (BHD) on the nucleation, growth kinetics, and micromechanical properties of eNiCo alloys was found to be the least investigated parameter.

Qiao et al. [27,28,29] reported the role of BHD (i.e., using high-speed electrolyte flow) on the grain size of eNiCo alloys, wherein they reported circa 20% grain size reduction with an increment in electrolyte jet speed from 250 m/min to 500 m/min [27]. However, the underlying influence on the grain refinement (i.e., the nucleation and growth behavior) was not a part of their study and, moreover, the implications of BHD flow rate on the micromechanical properties were also not reported. Gomez et al. [30] used a rotating disk electrode (RDE) as a mode of BHD, and reported that a uniform alloy composition along the depth of the deposit could only be obtained by using BHD, and at the same time they reported that it prevented the co-deposition of metal-hydroxides; however, the study was limited to depositions at 0 rpm and 1000 rpm, and the underlying influence on the nucleation behavior and micromechanical properties was not investigated. Bakhit et al. [31] reported that BHD resulted in homogenous alloy composition in the coating and increased the degree of anomaly; however, its influence on the micromechanical properties was not reported. Recently, Hu et al. [32] investigated the influence of BHD on the corrosion resistance of eNiCo alloys by employing BHD using the so-called large centrifugal acceleration mode, and reported an improvement in corrosion resistance and also, partly, the microhardness value (circa 460 HV to 530 HV for 0 rpm to 900 rpm). However, the microhardness value decreased with the further increase in the BHD rotation rate, wherein the improvement in the microhardness was attributed to the alloy cobalt content Co_A_. However, the influence on nucleation and growth behavior was not investigated and, additionally, the proposed setup with cylindrical cathode could not be used for the microforming using LIGA technique (which is done on flat substrates [1]).

Therefore, in comparison to above mentioned ECD operating conditions, the role of BHD, considering its potential and prospects towards fabrication of nC-deposits, according to the literature review, is the least investigated parameter for the eNiCo alloy system. Moreover, considering the above discussion and the summary reported by the authors of this paper in [14], it is obvious that the eNiCo alloy system still needs an extensive investigation with respect to the influence of BHD on the degree of anomaly, the resultant microstructure, and the respective micromechanical properties, in order to exploit its mechanical properties to a higher extent.

Hence, this work investigates exclusively the effects of BHD, realized by a magnetic stirrer, on the growth kinetics in the case of the eNiCo alloy system. The flow characteristics of the vortex created by magnetic stirrer is simulated, and an optimum region within the vortex flow is estimated with minimum variation in the vortex velocity. Chronopotentiometric deposition is performed with varying BHD conditions for deposition of eNiCo and eNi coating samples, to comprehensively investigate the influence of BHD in this alloy system. The deposited coatings are characterized by SEM, AFM, XRD, XRF, and Vickers microhardness to characterize the material related properties. Furthermore, the consequences of BHD on the nucleation and growth kinetics are modelled using the electrochemical phenomenon considering the reduction in the Nernst diffusion layer thickness δ as a function of BHD, and the physical phenomenon considering the fluid-cathode interaction as a function of BHD.

## 2. Materials and Methods

### 2.1. Electrochemical Deposition Experimental Setup

**Figure 1:**
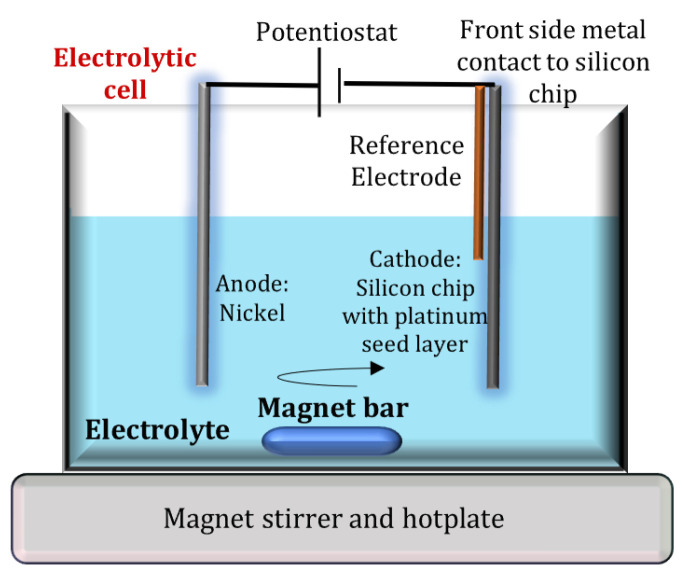
Schematic of conventional electrolytic cell with 3 electrodes, consisting of silicon coated with platinum plating base as cathode, nickel as anode, and Ag/AgCl as reference electrode. The bath hydrodynamics is induced with a magnet bar stirred by a magnetic stirrer.

A conventional electrolytic cell with three vertically aligned electrodes was used for the experiments with an electrolytic cell diameter of 10 cm, as shown in Figure 1. The anode consisted of dissolvable Ni plate (99.9% pure, 10 × 10 mm^2^ open area, 800 µm thick), while the cathode was made from (100) monocrystalline silicon wafer, sputter coated (using magnetron sputtering system, Alcatel SCM650, France) with 20 nm thick titanium, serving as an adhesion promoter, and a 100 nm thick platinum plating base. The wafer was diced into 10 mm × 40 mm large chips, of which a 10 mm × 10 mm area was dipped into the electrolyte for deposition. The electrodeposition of eNi and eNiCo alloy samples was carried out from freshly prepared additive free sulfamate bath. Analytical grade nickel sulfamate and cobalt sulfamate solutions (from Subolab GmbH, Pfinztal, Germany) were used for all the experiments. Boric acid was used as the buffering agent to maintain the bath pH value, and deionized water (conductivity < 0.01 µS/cm) was used for the preparation of the electrolyte. The concentrations of the used electrolyte constituents and the used ECD operating conditions are shown in Table 1. The metal content of the nickel sulfamate (180 g/L Ni) and cobalt sulfamate (30 g/L Co) solution was analyzed using complexometric titration with ethylenediaminetetraacetic acid (EDTA) using murexide indicator. The Co content in the electrolyte Co_E_ used for the ECD of eNiCo alloy samples for the bath composition shown in Table 1 was circa 2 wt%. The pH of the bath was adjusted using sulfamic acid and sodium hydroxide solution, and controlled using Hana pH meter (HI 2211, Hanna Instruments Ltd., Bedfordshire, UK). The bath temperature was maintained at 45 ± 2 °C by heating the electrolytic cell on a hotplate integrated with stirrer (Phoenix Hotplate RSM-10HP, Phoenix Instrument, Garbsen, Germany), therefore, it also served in varying the BHD conditions with a magnetic stirring function (clockwise rotational direction) using a PTFE coated magnet bar (RSM-E 220: 30 mm length and 8 mm diameter). Ag/AgCl reference electrode from Princeton Applied Research (Model K0265, AMETEK Scientific Instruments, Oak Ridge, TN, USA) with 3 M saturated KCl solution was used as the reference electrode. The reference electrode was placed in a very close proximity to the cathode to ensure minimum ohmic loss. The electrochemical cell was covered using a polydimethylsiloxane lid to reduce the influence of evaporation of the electrolyte during the experiments. The ECD was carried out using a Potentiostat (Biologic SP150, Biologic, Seyssinet-Pariset, France) with EC-Lab interface (v11.30, Biologic, Seyssinet-Pariset, France).

### 2.2. Material Characterization

The electrodeposited alloy composition of eNiCo samples was characterized using X-ray fluorescence (XRF) spectroscopy (Ametek Spectro Midex, SPECTRO Analytical Instruments GmbH, Kleve, Germany) with a spot size of 1 mm (5 measurements were done for each sample), the deposition thickness was measured using a digital gauge from Carycompar Meseltron, the surface morphology was characterized using scanning electron microscope (SEM, JEOL 5400, JEOL Ltd., Tokyo, Japan) in secondary electron mode, the average surface roughness $R_{a}$ and the surfaces’ fractal dimension FD was characterized using Park Instruments atomic force microscope (AFM, Park Systems Corporation, Suwon, South Korea) with a scan surface area of 20 µm × 20 µm.

Vickers microhardness was measured using a 100 g load for coatings with thickness > 50 µm, and 10 g load for coatings with thickness < 50 µm, using Innovatest Falcon 501 Vickers Microhardness device (Innovatest Europe BV, Maastricht, The Netherlands). Each microhardness value is the mean value of 10 measurements made on each sample at different positions. Additionally, the measured microhardness values were correlated with the respective grain size d according to the Hall–Petch relationship of yield strength, and the relationship between yield strength and hardness, which holds for most materials, the measured hardness values as described in Equation (1).

(1) $$H={\sigma_{o}}^{\prime }+\frac{{k_{y}}^{\prime }}{\sqrt{d}\;}$$

where ${\sigma_{o}}^{\prime }\;$ and ${k_{y}}^{\prime }$ are material specific constants, derived, respectively, from the according constants $\sigma_{o}$ and $k_{y}$ for the Hall–Petch relation of yield strength. Thus, besides a term not depending on the grain size d , the *H* values are inversely proportional to the square root of d values. The X-ray diffraction (XRD) measurements were realized using Seifert 3003 HR (GE Inspection Technologies, Pennsylvania, USA) with Bragg–Brentano geometry with CuKα1/2 radiation alloys (STOE StadiVari goniometer equipped with an ultra-fast and sensitive DECTRIS PILATUS pixel detector (300 K) and two microfoci X-ray sources, i.e., Cu- and Mo-radiation) with X-ray wavelength 0.1540562 nm to characterize texture, and the preferred crystallographic orientation. The instrument broadening was, for all lines, approximately 0.06°, and all scans were done from 10–90° with 0.01°/s. All measurements have been realized with full illumination of the sample, wherein the mixed radiation CuHα1/2 is reduced by the Rachinger correction to a quasi-monochromatic measurement with CuKα1 with background reduction. The grain size d is computed from the recorded XRD diffractograms using the Debye–Scherrer’s relation (Equation (2)), where k is the Scherrer constant (taken as 0.94 considering spherical crystallites [33]), λ is the wavelength of X-ray used (i.e., 0.1540562 nm), β is the full width at half maximum (FWHM) of the diffractogram peak (in radian), and θ can be extracted from the  2θ (in radian) values for the respective peak occurring in the XRD diffractogram.

(2) $$d=\frac{k\lambda }{\beta cos\theta }$$

## 3. Modelling of Electrolyte Vortex Flow Created by Magnetic Stirring and Determination of Optimum Electrode Position within the Vortex

BHD conditions can be realized by several approaches, such as mechanical stirring, rotating disk electrode, nitrogen bubbling through the electrolyte, laminar electrolyte flow towards the cathode, ultrasonic agitation, etc. Although the realization of BHD by mechanical stirring, i.e., by magnetic stirrer or by motor driven propeller stirrer, results in difficult to model flow, owing to its inhomogeneous flow field across the electrolytic cell, it is still the most commonly used method in ECD to realize BHD [34,35]. Therefore, in this section, the electrolyte vortex velocity profile, realized by magnetic stirring, is initially simulated and, subsequently, the electrolyte vortex velocity is validated experimentally using a Pitot tube. Owing to the strong flow variations within the vortex across the electrolytic cell, an optimum electrode sample position in the resultant vortex is determined, at which the flow variations are minimum. Furthermore, it is verified experimentally by considering minimum variation in the alloy cobalt content Co_A_ for the samples placed at that optimum position as reference.

### 3.1. Simulation of the Electrolyte Vortex Flow Created by Magnetic Stirrer

**Figure 2:**
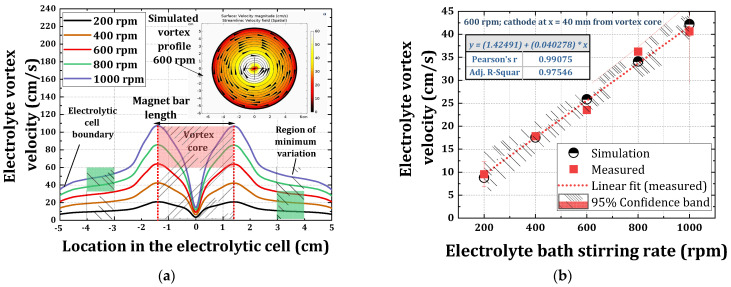
(**a**) Simulated 2D cross-sectional profile of the electrolyte vortex flow along the electrolytic cell, realized by a magnetic stirrer at varying bath stirring rates (inset shows the 2D simulated profile for 600 rpm, with top-view perspective of cell); (**b**) Simulated and measured electrolytic vortex velocity in stirred NiCo electrolyte as a function of varying bath stirring rates taken at distance of 40 mm (i.e., x = 40 mm) away from the vortex core.

BHD realized by magnetic stirrer is simulated using the computational fluid dynamics (CFD) module with mixer model of the COMSOL Multiphysics software (v5.2, Comsol Multiphysics GmbH, Göttingen, Germany). The boundary conditions defined for the simulation emulated the experimental conditions, which included: the electrolytic cell diameter (10 cm), the measured electrolyte density (1.2 kg/dm^3^), and the magnet bar dimensions (length: 3 cm and diameter: 8 mm). The material definition of the electrolytic cell was defined by quartz material, and the electrolyte definition was achieved by modifying the built-in water, liquid material from the materials library with respect to the density of used electrolyte. The simulations were carried out by varying the electrolytic bath stirring rates from 200 rpm to 1000 rpm with an interval of 200 rpm. For the simulation of the flow, the presence of thin electrodes (sub-millimeter thickness) was neglected. As an example, the 2D simulated surface profile plot of the electrolyte vortex created by magnetic stirrer at 600 rpm is shown in the inset of Figure 2a.

This surface color plot shows the stirred electrolyte’s convective velocity ( v ) in cm/s and the streamlines with arrows show the clockwise directional movement of the electrolyte. From the parameterized simulation study with varying stirring rates, it was found that the profile distribution of v is inhomogeneous across the electrolytic cell, with v being maximum in the center of the electrolytic cell and decreased towards the boundary of the electrolytic cell. This phenomenon can be well-explained using the cross-sectional profile of v along the electrolytic cell at different stirring rates as shown in Figure 2a. Here, the abscissa is the diameter of the electrolytic cell (x = 0 cm depicts the center of the electrolytic cell, which is also the magnet bar’s central axis of rotation) and the ordinate is v . Within the magnet bar’s axis of rotation (i.e., −1.5 cm < x < 1.5 cm), v increased steeply from the center of rotation, and reached a maximum at x = −1.5 cm and 1.5 cm, respectively. This corresponds to the edge of the magnet bar, where the maximum rotational force occurs, and v gradually decreased towards the boundary of the electrolytic cell where, initially a steep fall in v (i.e., 1.5 cm < x < 3 cm) is observed with circa 45% from its peak value (owing to the symmetry same trend is observed on the left side, i.e., for x = −1.5 cm and x = −3 cm). In addition, for 3 cm < x < 4 cm, v decreased by circa 10% and subsequently, decreased steeply again towards the cell (18% decrement, i.e., 4 cm < x < 5 cm). Moreover, at any given position in the electrolytic cell, the simulated v increased linearly with the bath stirring rates, as shown in Figure 2b, measured at x = 4 cm.

### 3.2. Measurement of Electrolyte Vortex Velocity

To physically derive the transfer rate from rpm to v , a self-made Pitot tube made out of transparent polyethylene with 4 mm inner diameter, 1 cm lateral tip length, and 15 cm total length was used for the measurements. For the measurements, the Pitot tube was placed in the electrolytic cell at a distance of 4 cm from the vortex core (i.e., within the region of minimum variation) with its opening placed tangential towards the vortex flow (i.e., aligned to the direction of the flow). Consecutively, the electrolyte entering the Pitot tube resulted in an increase in the level of the electrolyte in the Pitot tube, which is proportional to v as a function of the bath stirring rate. The generalized Bernoulli’s equation was used to calculate v using Equation (3), where μ is a dimensionless calibration coefficient, g is acceleration of gravity (i.e., 9.81 m/s^2^), and Δh is the rise in the electrolyte level within the Pitot tube’s column in response to the local velocity of the electrolyte (computed using Equation (4)), where $h_{stationary}$ and $h_{steady}$ are the electrolyte levels in the Pitot tube under stationary and BHD conditions, respectively.

(3) $$v=\mu \cdot \sqrt{2g\Delta h}$$

(4) $$\Delta h=h_{steady}-h_{stationary}$$

The bath stirring rate was varied from 200 rpm to 1000 rpm with an interval of 200 rpm, and the corresponding rise in the electrolyte level within the Pitot tube’s column (i.e., Δh) was recorded five times for each bath stirring rate. Subsequently, v was calculated using Equation (3) by considering μ as 1 [36], and plotted as a function of bath stirring rate as shown in Figure 2b, where the bath stirring rate from 200 rpm to 1000 rpm resulted in v (at 4 cm from the vortex center) from 8 cm/s to 42 cm/s, respectively. A good agreement is found between the simulated vortex velocity and the measured vortex velocity from the vortex core (Figure 2b). The computed values for v (i.e., 8 cm/s to 42 cm/s) are thus lower when compared to those reported in the literature using high-speed jet type of BHD, with v ranging from 250 m/min to 500 m/min (i.e., 417 cm/s to 834 cm/s), as reported Qiao et al. [27,28,29] for the ECD of NiCo alloy system.

### 3.3. Determination of an Optimum Electrode Position in the Electrolyte Vortex Flow

**Figure 3:**
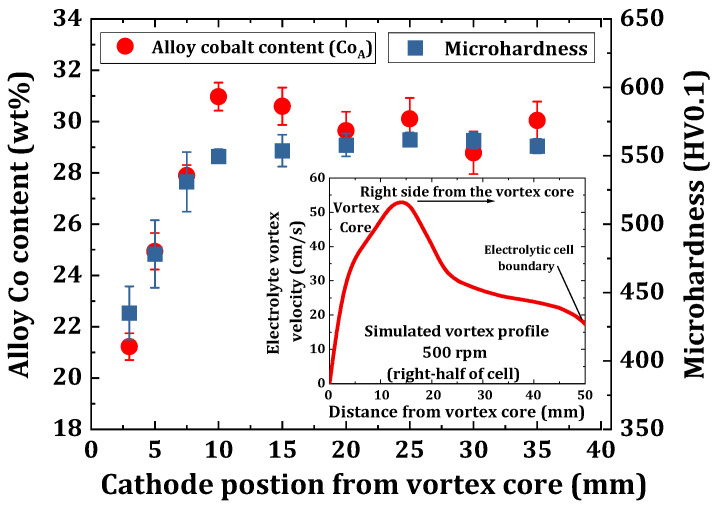
Influence of the varying electrolyte flow velocity in vortex on the alloy Co content and the resultant microhardness of eNiCo alloy samples, electrodeposited at 500 rpm, 50 mA/cm^2^, 45 °C, pH 4 for 1 h. The inset shows the simulated vortex profile (right-half) at 500 rpm for NiCo electrolyte.

In order to investigate the influence of BHD using a magnetic stirrer on eNiCo alloys, a vortex flow region within the electrolytic cell with minimum local variation in v has to be determined, where the electrodes can be placed during the experiments, resulting in reproducible results by avoiding abrupt change in  v . Hence, investigations were carried out by electrodepositing eNiCo alloy samples by placing the electrodes (i.e., nickel anode and silicon cathode) at different positions from the vortex core and characterizing the electrodeposited alloy for their respective alloy Co content, Co_A_, and microhardness values. Moreover, the anomalous co-deposition nature of eNiCo alloys (i.e., the less noble metal, Co being more preferentially co-reduced [14]) along with the fact of cathodic reduction of $Co^{2+}$ being controlled by mass transportation, also makes eNiCo alloys ideal candidates to investigate the implications of varying v across the electrolytic cell, as the Co_A_ content is highly sensitive to the variations in v . Therefore, the region of minimum variation in v can be directly derived by measuring the Co_A_ content of the eNiCo coating samples electrodeposited at various locations across the vortex, and by selecting the samples showing a minimum variation in measured Co_A_ content would correspond to the region of minimum variation in v (Figure 3).

For the investigations with respect to varying vortex profiles across the cell, the electrodes were placed symmetrical with respect to the vortex core, and were moved equidistant for inter-electrode distances in the range of 5 mm to 70 mm. Chronopotentiometric depositions were carried out using the electrolyte and ECD operating conditions as shown in Table 1, at a stirring rate of 500 rpm and for a duration of 1 h. Figure 3 shows the sensitivity of the electrodeposition of eNiCo alloys to the changing v profile, where the measured Co_A_ content is seen to follow the variation in v , which is shown in the inset of Figure 3. There is circa 10 wt% absolute change in the Co_A_ content, corresponding to the abrupt rise in v between 0 mm and 15 mm cathode position from the vortex core. However, the Co_A_ content only decreased by circa 2 wt%, corresponding to the fall in v at 15 mm < x < 25 mm. Furthermore, at 25 mm < x < 40 mm, the gradual fall in v (i.e., ca 14%) had a relatively lesser influence on the Co_A_ content with < 2 wt% variation. As it is obvious that v decreases towards the cell boundary, which is also evident from the inset of Figure 3 with 40% variation in v at 40 mm < x < 50 mm, no depositions were done in this region. Hence, considering the minimum variation in  v (i.e., circa 7%) and a stable Co_A_ content at 30 mm < x < 40 mm, this region is selected for the experimental study and the anode and cathode were placed at x = −40 mm and x = 40 mm from the vortex core, respectively.

Additionally, the microhardness of the eNiCo coating samples was also measured, as shown in Figure 3. The measured microhardness followed a similar trend as the Co_A_ content as a function of the varying  v profile. It increased from 430 HV0.1 to 550 HV0.1 on moving from x = 0 mm to x = 15 mm, respectively, and it remained at a constant value of circa 560 HV0.1 on moving beyond x > 15 mm. In order to determine the nature of the vortex flow behavior, the Reynold’s number ( Re=ρvhη) was also recorded from the inbuilt function using the above simulations, where ρ is the density of the electrolyte, h is the characteristic height of the fluid flow in cell, and η is the viscosity of the electrolyte. As obvious from the above discussion, at a given stirring rate, $R_{e}$ varied across the electrolytic cell with the varying v . However, the area of minimum variation was used to record $R_{e}$ as a function of different stirring rates, where it varied from 130 to 660 for the stirring rates 200 rpm to 1000 rpm, respectively. Therefore, the flow range lies within the laminar flow regime for the investigated v range in this work.

## 4. Results and Discussion

### 4.1. Chronopotentiometric Electrodeposition

**Figure 4:**
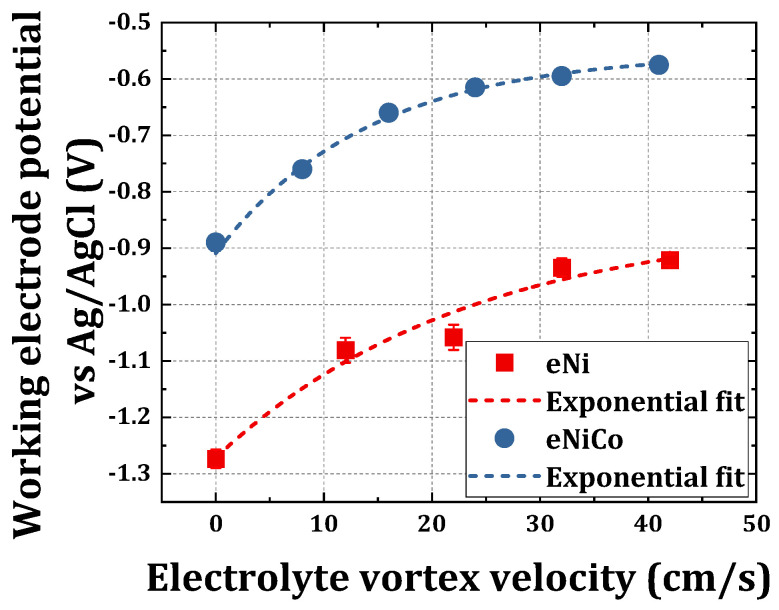
Working electrode potential $E_{WE}$ vs. Ag/AgCl reference electrode for j = 50 mA/cm^2^ as a function of BHD with varying velocity recorded during the chronopotentiometric analysis for eNi and eNiCo alloys with cathode placed at x = 40 mm from vortex core, bath pH value of 4, temperature 45 °C, averaged over entire deposition time of 15 min (eNiCo alloys) and 60 min (eNi).

In order to maintain a constant reaction rate during ECD, a chronopotentiometric (galvanostatic) technique was used in this study to deposit eNiCo and eNi coating samples to investigate exclusively the influence of BHD on the deposition kinetics and the resultant micromechanical properties. With respect to previous findings [37], a current density ( j) = 50 mA/cm^2^ was selected, as this was in the range of j , which resulted in eNiCo coatings with maximum microhardness for the electrolyte constituents used in this work (Table 1). As described in Section 3, the samples were electrodeposited by varying v from 0 cm/s to 42 cm/s, using the ECD operating conditions as listed in Table 1 for a duration of 15 min and 60 min for eNiCo and eNi coatings, respectively. The mean potential (average of recorded potential over the entire deposition duration) of the working electrode (WE) for j = 50 mA/cm^2^ was recorded with respect to the Ag/AgCl reference electrode as a function of varying v for both eNiCo coatings and eNi is shown in Figure 4.

For ECD without BHD, the potential $E_{WE}$ required for the deposition of eNiCo alloys is shifted by circa 400 mV compared to $E_{WE}$ for given current density for eNi coatings. This effect can be attributed to the presence of the $Co^{2+}$ ions in the electrolyte, which are more kinetically active compared to the $Ni^{2+}$ ions, thereby resulting in depositions even at a less negative $E_{WE}$ for eNiCo than for eNi, which is also reported by previous works [38]. Furthermore, an interesting trend of $E_{WE}$ change as function of v  is observed, wherein it is found to follow an exponential relation with increasing v for both eNiCo alloys and eNi coatings, as shown in Figure 4, using an exponential fitting line (with 0.96 and 0.99 coefficient of determination for eNi and eNiCo, respectively). The corresponding fit equations for eNiCo alloys and eNi coating samples are shown in Equations (5) and (6), respectively.

(5) $$E_{WE}\left(eNi\right)=-0.42\;\left[V\right]\cdot exp\left(-\frac{v\;\left[\frac{cm}{s}\right]}{23.2\;\left[\frac{cm}{s}\right]}\right)-0.85\;\left[V\right]$$

(6) $$E_{WE\;}\left(eNiCo\right)=-0.35\;\left[V\right]\cdot exp\left(-\frac{v\left[\frac{cm}{s}\right]}{14.1\;\left[\frac{cm}{s}\right]}\right)-0.55\;\left[V\right]$$

This behavior is related to the increase in mass transportation of the cations with increasing v towards the WE, which reduces the concentration gradient, and hence the needed $E_{WE}$ . However, a saturation in $E_{WE}$ is observed beyond v > 25 cm/s, which can be attributed to a process shifting to more activation limited whereby, even though there are enough free cations within the vicinity of the electrode, the number of charges, i.e., the free electrons on the WE surface, are limited by the controlled current technique (in this case 50 mA/cm^2^). The material characterization of these coatings is discussed in the proceeding sections.

### 4.2. Degree of Anomaly as a Function of Varying BHD Conditions

The anomalous co-deposition nature of eNiCo alloys makes the resultant alloy composition quite susceptible to changes in the ECD operating conditions. The degree of anomaly $d_{A}$ , given by Equation (7), represents the intensity of the anomalous co-deposition behavior, i.e., it is the factor for the excess of cobalt content in the electrodeposited alloy *Co_A_* as compared to its content in the electrolyte *Co_E_*.

(7) $$d_{A}=\frac{Co_{A}}{Co_{E}}$$

**Figure 5:**
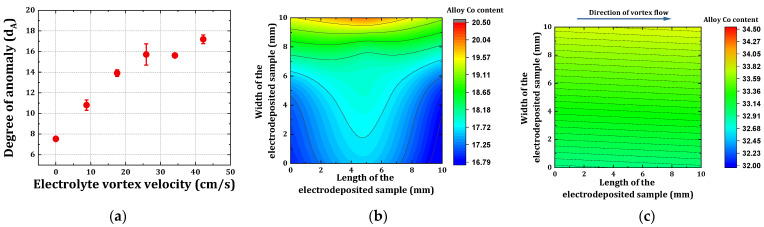
(**a**) Computed $d_{A}$ as a function of varying BHD conditions measured by XRF for eNiCo alloy coating samples electrodeposited at 50 mA/cm^2^, bath pH value 4, 45 °C for 15 min; (**b**) 2D contour plot showing the alloy mapping with the measured Co_A_ content (in wt%) across the coated surface for eNiCo alloy coating sample electrodeposited without BHD; and (**c**) eNiCo alloy coating sample with BHD at v = 42 cm/s. The cathode was placed at x = 40 mm from the vortex core for all depositions.

As reported earlier in our review paper [14], when all other ECD operating conditions are constant, the value of $d_{A}$ increased with increasing mass transport (e.g., by BHD) of the less noble species, i.e., $\;Co^{2+}$ , towards the WE surface. The eNiCo samples electrodeposited with varying BHD conditions (Section 4.1) were characterized by XRF to measure the respective Co_A_ content as listed in Table 2. Figure 5a shows the experimentally evaluated values of $d_{A}$ as a function of v by using the measured Co_A_ content using Equation (7). From Figure 5a, the anomalous co-deposition nature of eNiCo alloy is evident with $d_{A}$ = 7.5 for the samples electrodeposited without BHD. The anomaly degree $d_{A}$ increased first almost linearly with increasing v , i.e., it increased by a factor of circa 2 from $d_{A}$ = 7.5 to $d_{A}$ = 15.7 on increasing v from 0 cm/s to 25 cm/s, respectively. However, on increasing v > 25 cm/s, the rise in $d_{A}$ occurred with a lower slope, as also evident from Figure 5a. This behavior can be attributed to the mass controlled limited deposition nature of $Co^{2+}$ . The induced BHD by magnetic stirrer supplies the WE surface with fresh $Co^{2+}$ from the bulk of the electrolyte, which is preferentially cathodically reduced owing to anomalous co-deposition nature of eNiCo alloys. Furthermore, the rise in $d_{A}\;$ with a lower slope occurring at v > 25 cm/s can be attributed to the deposition reaching an activation limitation, as described above in Section 4.1, which is a function of j (discussed in Section 5.1).

The variation of the Co_A_ content across the surface of the eNiCo alloy coating samples (i.e., across the 10 mm × 10 mm surface of samples) was also investigated using XRF spectroscope, and Figure 5b,c show the derived contour plots for the eNiCo alloy samples electrodeposited without and with BHD ( v = 42 cm/s), respectively. With BHD, the uniformity distribution of alloy composition across the coating sample is improved (Figure 5c) when compared to the sample without BHD (Figure 5b). In the case of the coating electrodeposited without BHD; the Co_A_ content varied across the coating sample surface by circa 20% (relative change). Moreover, as shown by the contour lines corresponding to the distribution of Co_A_ content (Figure 5b), the effects of current density distribution, the concentration, and temperature gradients occurring on the surface of the WE without BHD are evident as a function of the Co_A_ content. Wherein, the current density concentration at the top edge of the electrode might enhance the Co_A_ content, leading to circa 20.5 wt% Co_A_. On the contrary, the contour plot (Figure 5c) for the eNiCo alloy sample electrodeposited with BHD ( v = 42 cm/s) shows a more homogenous distribution of the Co_A_ content, with only circa 3% relative change in Co_A_ content across the coating sample surface. This homogenizing effect can be attributed to the constant availability of the fresh $Co^{2+}$ at WE surface, and hence resulting in a homogenous distribution of alloy composition across the coating sample surface compared to a more inhomogeneous distribution without BHD. Furthermore, the contour lines still show small variations in the Co_A_ content across the coating surface, which may be correlated to the flow behavior, wherein the direction of the vortex flow was clockwise, thereby resulting in flow direction from the right to the left side of the investigated chip area.

### 4.3. SEM and AFM Surface Morphology Characterization

**Figure 6:**
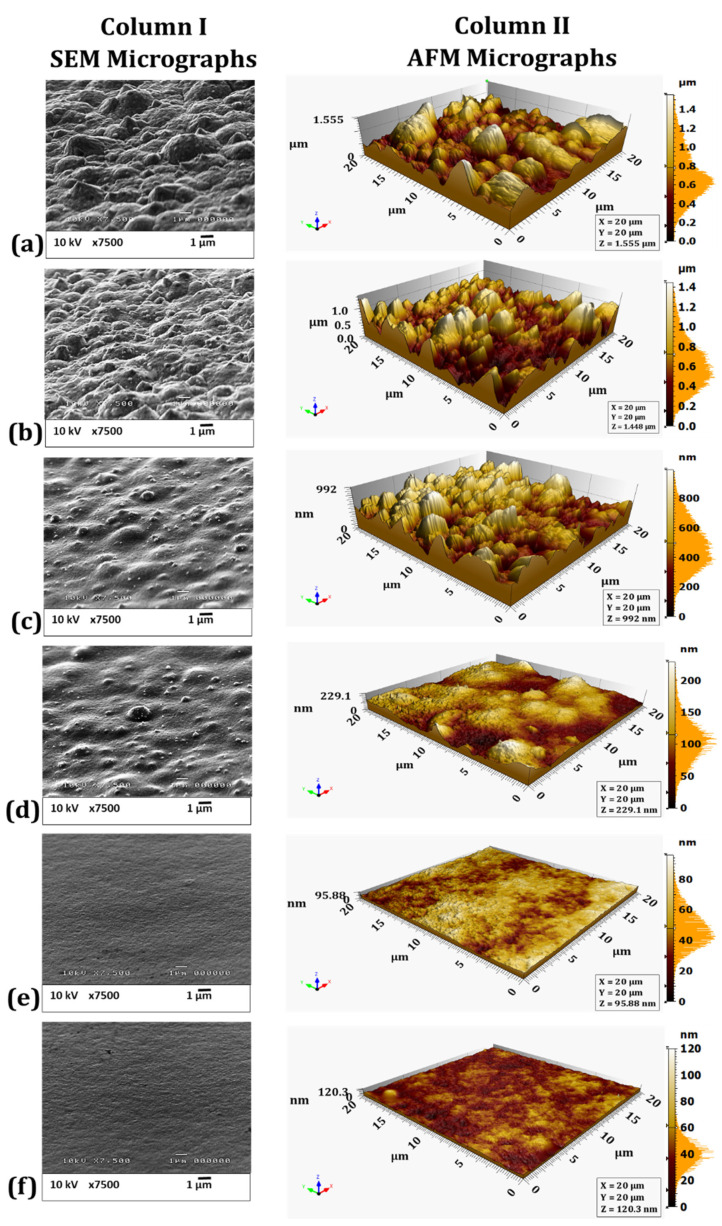
SEM micrographs with a magnification of 7500 (Column I) and AFM micrographs with a scan area of 20 µm × 20 µm (Column II) of eNiCo alloy coating samples electrodeposited at 50 mA/cm^2^, pH 4, 45 °C, 15 min with cathode at x = 40 mm from vortex core at different BHD by varying flow velocities *v*, (**a**) 0 cm/s; (**b**) 8.8 cm/s; (**c**) 18 cm/s; (**d**) 26 cm/s; (**e**) 34 cm/s; and (**f**) 42 cm/s.

**Figure 7:**
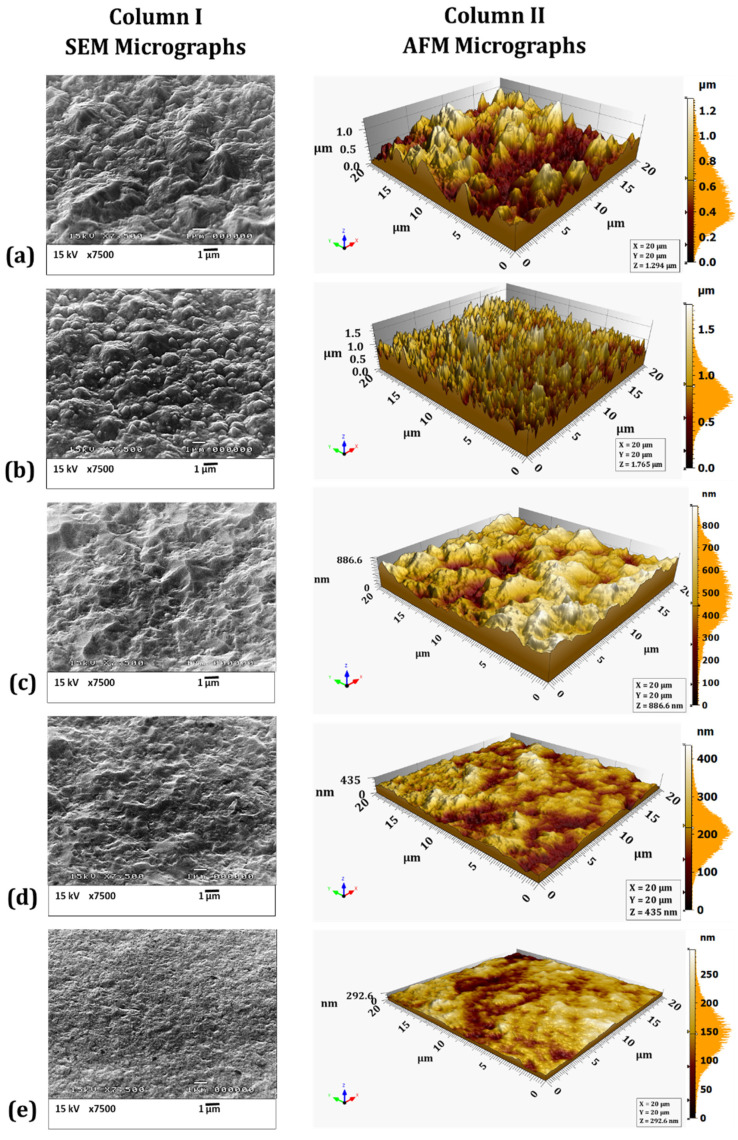
SEM micrographs with a magnification of 7500 (Column I) and AFM micrographs with a scan area of 20 µm × 20 µm (Column II) of eNi coating samples electrodeposited at 50 mA/cm^2^, pH 4, 45 °C, 60 min with cathode at x = 40 mm from vortex core at different BHD by varying flow velocities *v*; (**a**) 0 cm/s; (**b**) 12 cm/s; (**c**) 22 cm/s; (**d**) 32 cm/s; and (**e**) 42 cm/s.

**Figure 8:**
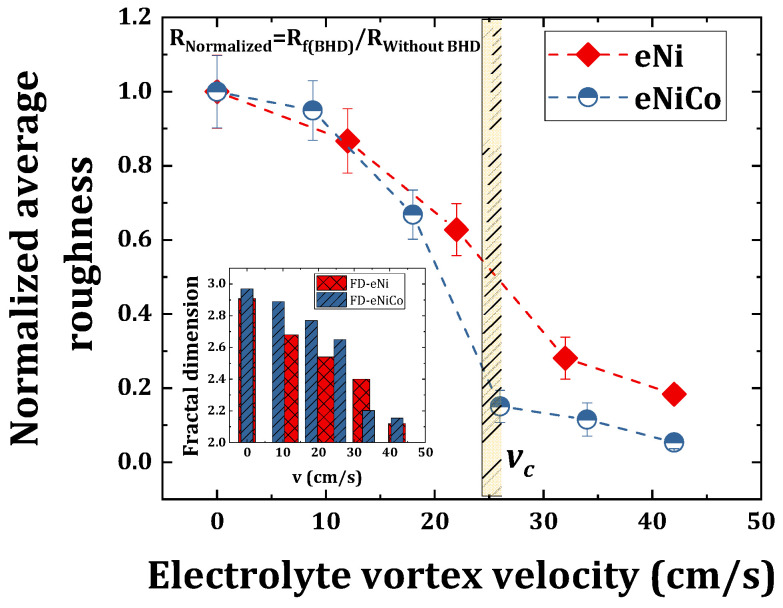
Average roughness values measured from the AFM micrographs for eNi and eNiCo alloy coating samples as a function of BHD with varying vortex velocities, electrodeposited at j = 50 mA/cm^2^, bath pH value = 4, bath temperature = 45 °C, and cathode position at x = 40 mm from vortex core. Deposition duration 15 min and 60 min for eNiCo coatings and eNi, respectively. Dotted line is shown to distinguish the data points. Absolute $R_{a}$ and FD values are listed in Table 2.

The surface morphology of eNiCo alloy and eNi coating samples was characterized by SEM and AFM, as shown in Figure 6a–f and Figure 7a–e, respectively. Apart from the qualitative surface morphology information obtained from the SEM micrographs, the average surface roughness ( $R_{a}$ ) was also computed from the AFM micrographs, and the mean $R_{a}$ of five measurements for each coating is summarized in Table 2. Furthermore, complementary to the $R_{a}$ values, the fractal dimension (FD) values giving an estimation of the character of surface morphology (more 3D for FD = 3, or 2D for FD = 2) were also computed. The computed FD values are also summarized in Table 2 and shown in the inset of Figure 8 as a function of v . As seen in Figure 6a, the surface morphology of the eNiCo alloy coating sample electrodeposited without BHD shows a columnar and granular cauliflower floret-like 3D surface morphology (due to the preferential orientation of growth towards applied electric field), with $R_{a}$ = 207.50 nm and FD = 2.97. However, for the eNiCo alloy coating samples electrodeposited with BHD, the surface morphology is found to become gradually smoother and more 2D with increasing v , which is evident from the SEM and AFM micrographs as shown in column I and II in Figure 6a–f, respectively and with the reduction in $R_{a}=f\left(v\right)$ as shown in Figure 8.

As summarized in Table 2, initially, with BHD at v = 8.8 cm/s, the granular growth behavior is slightly affected with circa 5% reduction in $R_{a}$ (Figure 8), with FD = 2.89 as shown in Figure 6b. However, the effect of BHD becomes prominent for velocity v > 8.8 cm/s, e.g., at v = 18 cm/s, a gradual suppression of the coarse granular growth behavior is observed (Figure 6c), which is further evident with a 33% reduction in $R_{a}$ value with FD = 2.77. Subsequently, for v = 26 cm/s, the suppression of the granular growth behavior is further enhanced with partial planarization (i.e., with occasional occurrence of suppressed granular growth) of the surface morphology (Figure 6d), with an enormous reduction of 85% in $R_{a}$ value with FD = 2.65.

Furthermore, on increasing v to 34 cm/s, the granular growth behavior is found to be suppressed to a higher extent, which is further evident by reduction of circa 88% in $R_{a}$ , and reduction in FD from 2.65 to 2.20 as compared to coatings deposited with v = 26 cm/s, as shown in the inset of Figure 8. Increasing v to 42 cm/s resulted in further planarization of the surface morphology, with a buffed shiny appearance of the coating, and a circa 95% reduction in the $R_{a}$ value with FD = 2.15, which signifies the extent of surface being confined to 2D surface morphology (Figure 6f).

Moreover, considering the slow planarization effect on the surface morphology with increasing v , and owing to the used geometry of the electrolytic cell, further increasing v > 42 cm/s caused spilling of the electrolyte (i.e., caused turbulent flow nature in the used electrolytic cell); therefore, the stirring rates > 1000 rpm were not used. Hence, from SEM and AFM characterization of the coating samples, it is found that the surface morphology of the eNiCo alloys with given fixed ECD operating conditions is significantly affected by exclusively varying the BHD conditions, wherein a smoothing of the surface morphology with increasing v is observed.

The SEM and AFM micrographs in column I and II in Figure 7a–e, respectively, show the surface morphology of eNi samples as a function of varying BHD conditions. In comparison to the cauliflower-like surface morphology for eNiCo coating sample without BHD (Figure 6a), the surface of eNi coating sample without BHD shows a pyramidal surface morphology oriented in the direction of the applied current field, with $R_{a}$ = 182 nm and FD = 2.91. With BHD at v = 12 cm/s, the pyramidal morphology is gradually hindered, which is evident from circa 13% reduction in $R_{a}$ value with FD = 2.70 (Figure 7b). Moreover, on further increasing v to 22 cm/s, the pyramidal surface morphology with sharp tips is further suppressed with partial planarization of the surface morphology, which is evident from circa 37% reduction in $R_{a}$ with FD = 2.5. On increasing v to 32 cm/s, further planarization of the surface morphology occurs (Figure 7d), which is depicted by circa. 71% reduction in $R_{a}$ value with FD = 2.40. Subsequently, on increasing v to 42 cm/s, the pyramidal nature is significantly hindered with circa 81% reduction in $R_{a}$ value with FD = 2.2, respectively.

Furthermore, pure Co coatings (eCo) were also deposited; however, as eCo coatings tend to peel off due to high internal stress in the coatings, the current density for eCo electrodeposition was reduced from j = 50 mA/cm^2^ to 10 mA/cm^2^. Irrespective of the lower j used, the surface morphology of the eCo coatings was found to undergo a certain degree of planarization as f(BHD) . For example, $R_{a}$ was found to decrease from 142 nm (FD 2.7) to 73.7 nm (FD 2.37) for eCo coatings electrodeposited without and with BHD (1000 rpm), respectively. Moreover, the difference in degree of planarization for eCo coatings can be attributed to the intricate relationship between BHD and other ECD operating conditions, therefore obviating that exclusively varying the BHD conditions significantly influences the nucleation and growth kinetics, both in the case of single element and in the case of alloys for the eNiCo alloy system. Furthermore, as shown in Figure 8, the effect of BHD on the surface morphology comes to a gradual saturation beyond critical velocity ( $v_{c}$ ), both in the case of eNiCo coatings and eNi coatings, which is explained with the help of a qualitative model in Section 5, and which also corresponds qualitatively to the critical velocity found for the degree of anomaly (Figure 5a).

### 4.4. X-ray Diffractograms, Growth Phase and Grain Size

**Figure 9:**
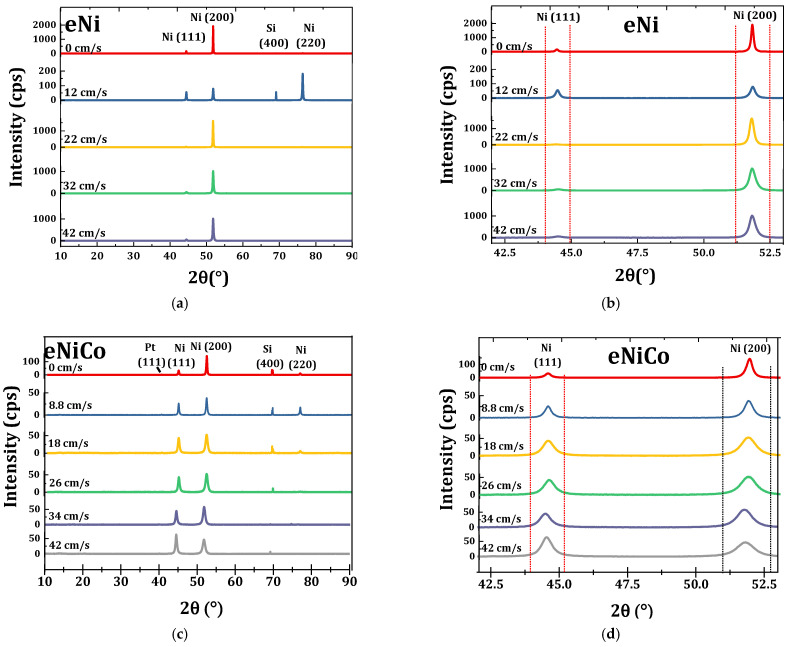
XRD diffractograms showing textured growth with predominant (200) and (111) Ni peaks as function of BHD; (**a**) eNi coating samples (scanning range 10–90°); (**b**) Magnified view (42–53°) of the predominant peaks of eNi coating samples; (**c**) eNiCo alloy coating samples (scanning range 10–90°); and (**d**) Magnified view (42–53°) of the predominant peaks of eNiCo alloy coating samples.

Figure 9a,c show the XRD diffractograms (10–90°), as a function of varying BHD for the eNi and eNiCo alloy coating samples, respectively. The diffractograms show a face-centered cubic (fcc) phase for both eNiCo and eNi coating samples.

The recorded predominant peak’s full width at half maximum (FWHM) is listed in Table 2, where it is found that a peak-broadening occurs as a function of increasing  v . Furthermore, the diffractogram of eNi samples (Figure 9a) shows the growth predominant in (200) orientation, while secondary peaks corresponding to the diffractions from (111) and (220) planes of eNi coatings and (400) peak from the silicon substrate are also recorded. However, these secondary peaks from the coatings are found to get suppressed as a function of v , wherein the weak (220) peak has no measurable intensity at v > 32 cm/s, and the intensity of Ni (111) peaks is reduced by circa. 65%, i.e., from 161 cps to 56 cps on increasing v from 0 cm/s to 42 cm/s, respectively. Additionally, the peak-broadening effect as a function of increasing v is shown in the diffractogram in the range of 42–53° (Figure 9b) with (111) and (200) peaks.

In the case of eNiCo alloy coatings, the XRD diffractogram (Figure 9c) primarily showed the solid solution nature formed on alloying Co with Ni, which is evident from the presence of single fcc phase. This can be explained from the phase diagram of the NiCo alloy system, wherein the primary fcc phase occurs for Co_A_ content up to 65.5 at%, beyond which the alloy crystallizes with predominant hexagonal closed pack (hcp) phase [39,40]. As described in Section 4.2, and listed in Table 2, the Co_A_ content in the eNiCo alloy coating samples varied in the range of 15.1–34.4 wt% and, hence, the XRD diffractograms showed the fcc phase corresponding to Ni-rich alloys. Secondly, similarly to eNi coating samples, the diffraction peaks corresponding to (111), (200), and (220) planes for eNiCo coatings and (400) plane for silicon substrate were recorded as seen in Figure 9c. However, in comparison to eNi coatings, the (111) grain growth plane was not suppressed as a function of v , which can be attributed to the modification of the nucleation and growth kinetics by the presence and inclusion of Co during the cathodic reduction with enhancement of the growth in both (111) and (200) orientations. Additionally, for eNiCo samples, a peak broadening is found for the (111) and (200) peaks as a function of v as shown in Figure 9d.

**Figure 10:**
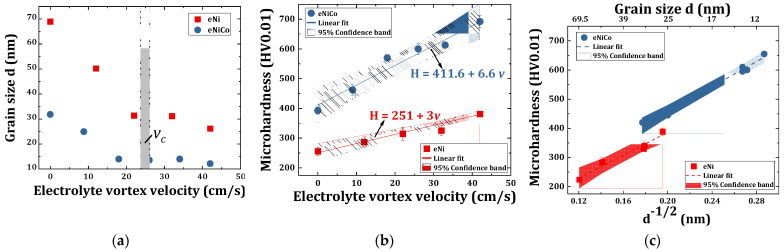
(**a**) Grain size computed from the predominant peaks from XRD diffractograms using the Debye–Scherrer relation for eNi and eNiCo alloy coating samples as a function of BHD; (**b**) Measured microhardness as the function of electrolyte velocity; and (**c**) Measured microhardness as the function of inverse square root function of the grain size, which fits well with the Hall–Petch relationship for eNi and eNiCo alloy coating samples.

The corresponding computed d values using Equation (2) for eNi and eNiCo alloy coating samples as f(BHD) are shown in Figure 10a and listed in Table 2. It can be seen that d  decreased for both eNi and eNiCo alloy coating samples as a function of BHD with increasing v . In the case of the eNi coatings, there is an initial 56% steep decrement in d from 70 nm to 31 nm on increasing  v from 0 to 22 cm/s, respectively. Subsequently, a critical velocity ( $v_{c})\;$ is observed, beyond which no significant reduction in  d is obtained, i.e., in this case, it occurs for v > 22 cm/s (Figure 10a). One aspect which needs to be considered is that the larger grain agglomerates in the surface morphology in the SEM and AFM micrographs in Figure 6 and Figure 7 does not reveal the grain size. This is especially observed in the case of ECD nC-materials, where the electrochemically deposited nC-materials tend to show larger grain agglomerates with rougher surface morphology [31,41,42,43]. For example, Bigos et al. [43] recently reported the nC nature ( d in the range 10–16 nm) of eNi coatings revealed by TEM characterization, even though $R_{a}$ values of the eNi coatings varied in the range of 75 nm to 190 nm, with surface morphology showing larger grain agglomerates.

Furthermore, in the case of eNiCo alloys coating samples, d also decreased as a function of increasing v . However, a very interesting effect of alloying Co with Ni on the resultant d for the eNiCo alloy coatings is observed, wherein in comparison to eNi coatings and irrespective of BHD conditions, d reduced by circa 50%. For example, it reduced from 70 nm for eNi coating (without BHD) to 31 nm for eNiCo alloy coating (without BHD), which can be attributed to the alloying effect, i.e., the presence of $Co^{2+}$ significantly affects the nucleation and growth kinetics, thereby resulting in the grain-refinement, i.e., reduction in the grain size for the resulting eNiCo alloys in accordance with [38,44]. Furthermore, as compared to eNi coatings, a similar trend in d vs. v is observed, wherein d reduced initially steeply by 56%, i.e., from 32 nm to 14 nm on increasing v from 0 cm/s to 18 cm/s, and with further increment in v , the reduction in d reached a gradual saturation.

This indicates that BHD conditions significantly affect the nucleation and growth kinetics, in the case of both eNiCo and eNi coatings up to $v_{c}$ , and beyond which the influence of BHD tends to gradually decrease, i.e., the growth process tends to be independent of further increase in $v>v_{c}$ . This behavior of grain refinement as f(BHD) can be attributed to electrochemical and physical effects of BHD as explained in Section 5.

### 4.5. Microhardness Characterization of eNi and eNiCo Alloy Samples

The Vickers microhardness values (*H*) of the eNi and eNiCo coating samples are listed in Table 2 and are represented as a function of v and the inverse square root of d (for comparison to Hall–Petch relation found for yield strength) in Figure 10b,c, respectively. The measured *H* values increased as a function of v for both eNiCo and eNi coating samples. In the case of eNi coating samples, hardness *H* increased by circa 49%, i.e., it increased from 255 HV0.01 to 381 HV0.01 on increasing v from 0 cm/s to 42 cm/s. Moreover, in the case of eNiCo alloy coating samples, the measured hardness of coating without BHD was significantly greater (i.e., by factor 2) than the eNi sample electrodeposited without BHD, which is directly attributed to the solid solution strengthening effect and grain-refinement effect by alloying Co atoms in the Ni lattice. Thereby, resulting in strengthening and an enhancement of *H* value. Furthermore, the *H* values of eNiCo coatings increased by circa 76%, i.e., it increased from 393 HV0.01 to 692 HV0.01 with increasing v from 0 cm/s to 42 cm/s. Thus, the hardness values for eNiCo show a slightly larger dependence on v than those of eNi. The measured *H* values, as described by Gilman [45], give an estimation of the degree with which solids can refrain from being plastically deformed in response to an externally applied load, which is directly related to hindering the propagation, proliferation, and interaction of the dislocations within the crystal lattice, wherein, among other phenomenon (reported in our previous paper [14]), the presence of smaller d results in a large grain boundary area within a given volume, which is an effective way to hinder the propagation of dislocations, is more predominant for nC-materials [21]. Hence, in order to correlate the influence of BHD conditions on the resultant micromechanical behavior of the resultant coating samples for the eNiCo alloy system, the *H* values are correlated with the computed d values according to the Hall–Petch relationship (Equation (1)). Figure 10c shows the change in the measured *H* values for both eNi and eNiCo coating samples as a $f\left(1/\sqrt{d}\right)$ , which fits well (having a narrow confidence band) with the Hall–Petch relationship.

Therefore, it is clear that the induced BHD conditions affected the nucleation and the growth kinetics by increasing v significantly, which is evident from the reduction in d as function of v . Hence, the grain-refinement effect predominantly resulted in the enhancement of the micromechanical properties of the resultant coatings of the eNiCo alloy system. However, a very interesting effect is observed for eNiCo samples electrodeposited with BHD at 18 cm/s and 34 cm/s, which resulted in similar d (Table 2), however measured *H* values differed. This effect can be attributed to the different Co_A_ content which simultaneously aids in the strengthening of eNiCo alloys.

Furthermore, yet another interesting observation can be made from Figure 10b, wherein the effect of BHD is found to be more prominent in the case of eNiCo coating samples, which is evident from a larger slope of 6.6 HV/(cm/s) of linear fit than the eNi coatings with a slope of 3 HV/(cm/s). This effect can be attributed to the anomalous nature of eNiCo alloy system, where $d_{A}$ increased with v (as discussed in Section 4.2), thereby resulting in a larger slope of the curve in *H* vs.  v curve than for pure eNi.

## 5. Model for the Influence of BHD on Nucleation, Growth Kinetics and Micromechanical Properties

Several theories and models are proposed in the literature to qualitatively justify the influence of BHD on nucleation and growth kinetics in the case of ECD. For example, Hussain [46,47] employed a jet speed mode of BHD, and considered the concept of continuous inflow of cations being instantly cathodically reduced as fresh nuclei at random sites, rather than diffusing and attaching to the already formed stable nuclei. Thereby, resulting in an enormous nucleation rate compared to ECD without BHD with finer grain size in nC regime, while Hu et al. [32] used a centrifugal acceleration system as a BHD mode for the eNiCo alloy system, and proposed that the limiting current density ( $j_{l}$ ) increased as function of v , which is apparently correlated to the increase in the used working electrode potential $E_{WE}$ for a given current density j , thereby resulting in enhanced nucleation probability W given by Equation (8), where *b* and *B* are constants.

(8) $$W=B\cdot exp-\left(\frac{b}{E_{WE}^{2}}\right)$$

Contrary to the results from Hu et al., considering the preceding discussion in Section 4.1, the chronopotentiostatic voltammograms revealed that, although $E_{WE}$ decreased as function of v , it resulted in reduced grain size (Figure 10a), which is evident from the SEM, AFM, and XRD characterization of eNiCo alloys and eNi coating samples (as discussed in Section 4), thereby indicating that the role of BHD in the reduction in grain size is a synergistic influence of several aspects, which needs to be considered. The primary aspect that needs to be considered is the enhancement of the mass transportation of the species from bulk of the electrolyte towards the cathode surface. This effect results in a consequent reduction in the diffusion layer thickness δ due to a reduced concentration gradient, subsequently enhancing the limiting current density $j_{l}$ , therefore this aspect of BHD can be considered as the electrochemical effects of BHD, which is discussed in Section 5.1. Secondarily, a phenomenon that has hardly been considered to date is the physical effects of BHD, i.e., the shear force ( $F_{s})$ exerted by the moving electrolyte on the cathode surface, which has a subsequent influence on nucleation and growth kinetics, and is discussed in Section 5.2.

### 5.1. Electrochemical Effects of BHD on Nucleation and Growth Kinetics during ECD

The electrochemical reduction of a given species at the cathode surface is synergistically controlled by the thermodynamics, the electrode kinetics, and the electrolyte kinetics. According to the classical description of mass transportation in ionic systems, the electrochemically active species are transported towards their respective electrodes by the principle phenomenon of migration (driving force being the electric field), diffusion (driving forces being the concentration gradient), and convection (driving force being hydrodynamic transport) as shown by the Nernst–Planck relation for 1D mass transport along x-axis in Equation (9), where the first, second, and third term correspond to the mass transportation by diffusion, migration, and convection, respectively.

(9) $$J=-D\;\frac{\partial c}{\partial x}-\frac{nF}{RT}\cdot D\cdot c\frac{\partial \varphi }{\partial x}+cv$$

In Equation (9), J is the flux of electrochemically active species (mol/s.cm), D is the diffusion coefficient (cm^2^/s), ∂c/∂x is the concentration gradient, ∂φ/∂x is the potential gradient, c is the concentration (mol/cm^3^), v is the hydrodynamic velocity (cm/s), n is the charge number of the electroactive species, F = 96,485 C/mol, the Faraday constant, R = 8.3145 J/mol.K, the gas constant, and T the absolute temperature (K). Furthermore, the diffusion velocity $\left(v_{d}\right)$ and the drift velocity $\left(v_{e}\right)$ of the electroactive species under the influence of the chemical and electrical potential are given by Equations (10) and (11), respectively [48].

(10) $$v_{d}=\frac{DF_{d}}{RT},\;where\;F_{d}=-\frac{RT}{c}\cdot \frac{dc}{dx}$$

(11) $$v_{e}=u.\left|E\right|,\;where\;u=\frac{n.e}{6\pi \eta a}$$

In Equation (10), $F_{d}$ is the driving force under concentration gradient dc/dx . In Equation (11), u is the mobility of the ions in m^2^/V.s, e is the elementary charge, η is the viscosity of electrolyte in (kg/m.s), and a is the hydrodynamic radius of the ions. From the electrolyte kinetics point of view, the use of BHD significantly increases the mass transportation of the cations towards the cathode. From Equations (10) and (11), the diffusion velocity $v_{d}$ = $17.3\times 10^{-3}$ cm/s and the drift velocity $v_{e}$ = $0.53\times 10^{-4}$ cm/s, (using D = 6.27 × 10^−10^ m^2^/s [49], η = 1.124 × 10^−3^ kg/m.s [50] and c from Table 1) are obtained for the experimental conditions used in Section 4. This shows that v as a function of BHD used in this work (i.e., 8–42 cm/s) is three and four orders of magnitude greater than $v_{d}$ and $v_{e},$ respectively, thereby indicating that the convective term in Equation (9) is predominant for the mass transportation. Hence, this controls the rate of reaction, and beyond that also influences the nucleation and growth kinetics, which is exemplified by the preceding discussion in Section 4. Consequently, with the enhanced mass transportation by BHD, a steady state condition is obtained with a linear decrement in concentration within the distance δ from the cathode surface, the resultant current density j can be expressed using Equation (12), where $c_{b}$ and $c_{c}$ are the bulk concentration and cathode surface concentration of the electrochemically active species, respectively [12].

(12) $$j=\frac{nFD\left(c_{b}-c_{c}\right)}{\delta }$$

Furthermore, as the reaction becomes limited by the mass-transport at larger polarization potential of the cathode, *j* no longer increases with increasing potential. Thus, although there exists an excess of charges at the cathode (by increasing polarization), due to an assumed constant concentration gradient at cathode, there occurs no further increase in deposition rate. Thus, a saturation in j occurs. This current density, which is independent of increasing potential, is termed as limiting current density $j_{L}$ given by Equation (13), wherein $c_{c}$ = 0 is assumed, as all the cations are immediately reduced upon reaching the cathode surface [12]. Moreover, in the case of a mass transport limited process, the value of $\;j_{L}$ can be enhanced by decreasing δ , as shown in Equation (13). Therefore, by increasing the intensity of applied BHD, the mass transportation of electroactive species can be further increased, δ can be decreased, and, consecutively, the value of $\;j_{L}$ could be elevated.

(13) $$j_{l}=\frac{nFDc_{b}}{\delta }$$

With an onset of cathodic reduction, there is a gradual depletion of cations in the vicinity of cathode, which results in a build-up of concentration gradient which determines the thickness of δ . However, in the case of ECD without BHD, the concentration gradient increases as a function of deposition time given by Equation (14) [12], and hence results in an increase in δ during deposition.

(14) $$\delta =\sqrt{\pi Dt}$$

**Figure 11:**
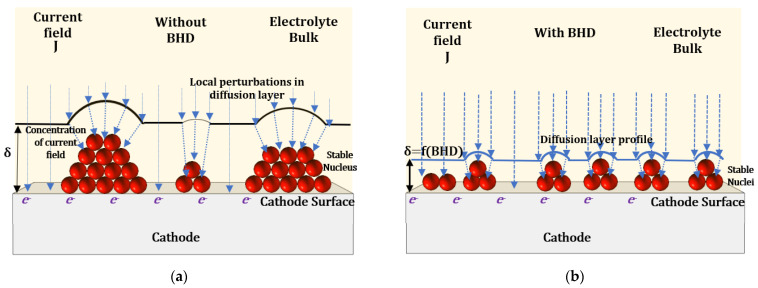
Schematic showing local perturbation in diffusion layer during the nucleation and growth in ECD of eNiCo alloy system; (**a**) without BHD, with its respective diffusion layer profile and concentration of the electric field on the growing nuclei, where a large gradient locally builds up between the larger size nucleus and the smaller size nucleus; and (**b**) with BHD, along with its diffusion layer profile. The dashed blue lines represent the electric field lines and thus the current direction.

In the case of ECD with BHD, there is a constant supply of electroactive species towards the cathode; consecutively, the concentration gradient within the vicinity of the cathode is reduced, and, hence, δ is reduced with a steady maintained (lower) concentration as function of time [51]. As reported by Eliaz and Gileadi [12], δ was computed to be in the range of 150 µm, 50 µm, 30 µm, and 2 µm for electrodeposition without BHD, with BHD using magnetic stirring, rotating disk electrode (400 rpm) and impinging jet, respectively (for $n=2,\;D=6\times 10^{-6}$ cm^2^/s). Furthermore, in this case, the thickness δ is determined by velocity of BHD, i.e.,  δ=f(BHD) . The implications of changes in δ as f(BHD) on nucleation and growth kinetics are schematically depicted in Figure 11a,b.

For the eNiCo alloy system, the case of 3D nucleation (i.e., adatoms reducing around a growing/stable nucleus in all three dimensions) with diffusion limited growth with unoriented dispersion type, as described by Winand [52], is considered. Furthermore, ECD without additional additives and levelling agents is assumed. In the case of ECD without BHD, as shown in Figure 11a, initially, owing to larger  δ , the nucleus growth is promoted, and secondarily the diffusion layer deforms in front of the formed nuclei, complying with the growth of nuclei [53]. Consecutively, this results in a pronounced concentration of the electric field lines at the peak of the growing nucleus without BHD, thereby facilitating the reduction in adatoms on it, while the freshly formed smaller nuclei at the base either grow at a slower rate due to the formed electric field gradient, or might merge with the strong growing nucleus by further diffusion to appropriate sites of the stable nuclei and further increase their size: the end result would be a deposited layer with coarse grain size. This is further evident from the preceding SEM and AFM results (Section 4.3), where the coatings without BHD resulted in coarse surface morphology, as expected for Volmer–Weber type nucleation mechanism. Additionally, this phenomenon is confirmed by Harniman et al. [53], who reported quantitatively the subtle evolution of the hydration layers associated with growing nuclei with advanced in situ measurement approaches, which can be correlated to the occurrences of the local gradients in the current density for the growing nuclei.

On the contrary, in the case of BHD, as discussed earlier, δ decreasesd with  v , therefore, as shown in Figure 11b, the local “deformation” in the diffusion layer, due to already grown nuclei, is smaller than without BHD and, moreover, the electric field gradient between the growing nucleus and freshly formed nucleus is smaller, thereby facilitating the formation of fresh nuclei rather than promoting grain growth: the end result is, therefore, a deposit with finer grain size, i.e., it results in grain growth in the nC regime. Subsequently, the layer growth, i.e., perpendicular to the cathode surface, occurs as a function of steady nucleation and growth process as function of BHD.

The implications of reduction in δ as function of BHD on the nucleation and growth kinetics was modelled in situ by Hyde and Crompton [34] as shown in Equation (15), where δ is related to the nucleation rate ( A), i.e. , the number of nuclei formed per active site/second, and the description of variable B can be found in [34]. The relation was used to extract A by fitting the recorded potentiostatic voltammograms (i.e., current-time transients) obtained using varying BHD conditions. Additionally, their model was reported to be applicable for the different modes of realizing BHD, such as stirred bath, flow cell, and ultrasonics.

(15) $$\delta =nFc\;\frac{D}{j}\;\left(1-exp\left\{-B\sqrt{t}\left[1-e^{-At}\overset{\infty }{\underset{n=0}{\sum }}\frac{{\left(At\right)}^{n}}{n!\left(2n+1\right)}\right]\right\}\right)$$

Moreover, with the use of a rotating disk electrode to initiate BHD during ECD, the consequences of BHD on δ has been extensively modelled and given by the Levich equation (Equation (16)), where ω is the angular velocity and *η* is the viscosity of the electrolyte. The approach is an excellent technique for the in situ analytical modelling of steady-state reduction kinetics; however, it is difficult to characterize the micromechanical properties of deposited coatings [12].

(16) $$\delta =1.61\;D^{1/3}\omega^{-1/2}\eta^{1/6}$$

Furthermore, in the case of BHD with magnetic stirring, the relation v created by BHD and δ could be obtained by recording the $j_{l}$ values with varying BHD (Equation (17)), for example, using hydrodynamic linear sweep voltammetry (HLSV) technique and solving the equation for δ as shown in Equation (18).

(17) $$j_{l}=f\left(v\right)$$

(18) $$\delta =\frac{nFDc_{b}}{j_{l}\left(v\right)}$$

**Figure 12:**
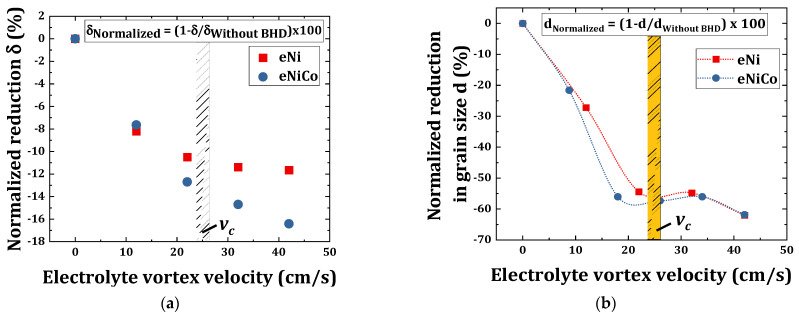
(**a**) Normalized diffusion layer thickness δ as function of BHD for the ECD of eNiCo and eNi computed from hydrodynamic linear sweep voltammetry in situ technique computed using Equation (18). The analysis was carried out using the electrolyte and ECD operating conditions composition as listed Table 1 with cathode being placed at x = 40 mm from the vortex core; and (**b**) Normalized grain size of eNiCo and eNi coating samples as a function of BHD, the dotted line is shown to distinguish the grain size reduction in the case of eNiCo and eNi coating samples.

In order to compare this model with the experimental results, HLSV with voltage sweep from 0 V to −1.2 V, with a sweep rate of 20 mV/s, was performed and the respective $j_{l}$ values occurring at −1.2 V were recorded. Consecutively, δ was computed by considering $c_{b}$ , as described in Table 1, and D taken from [49]. The detailed description of the electroanalytical in situ modelling including HLSV for the eNiCo alloy system as a function of BHD will be published elsewhere. Figure 12a shows the normalized computed δ for eNi and eNiCo alloys wherein, interestingly, δ initially decreased steeply to $v_{c}$ , i.e., it decreased by 10% for ECD of eNi and by 13% for ECD of eNiCo alloys. Furthermore, for BHD beyond $v_{c}$ , it decreased by 2% and 4% for eNi and eNiCo alloys, respectively.

Additionally, Figure 12b shows the normalized reduction in grain size of eNiCo and eNi coating samples as function of varying BHD conditions. Comparing Figure 12a,b, the grain size d seems to follow a similar trend as a function of BHD as δ , where it decreased by circa 55% on increasing the BHD up to $v_{c}$ , and this trend gradually slowed down with circa 6% reduction in d with an increase in BHD > $v_{c}$ up to 42 cm/s. The initial steep reduction in δ and d as a function BHD up to $v=v_{c}$ can be attributed to the electrochemical effect as described above, wherein BHD influences δ , which consecutively influences the nucleation and growth kinetics.

However, it has to be noted that the nucleation and growth kinetics are also governed by the physical aspects (Section 5.2), therefore the trend in Figure 12a,b are not exactly identical to one another. Furthermore, the difference in the scale of y-axis is obvious from Figure 12a,b, which reveals that the change in δ has a larger impact on resultant d , e.g., at BHD = 10 cm/s, the reduction in d is by factor 3 greater than the reduction in δ . For example, Hyde and Crompton [34] reported, through electrochemical analysis, the rate of nucleation increased by an order of magnitude from 10^2^ nuclei/s to 10^3^ nuclei/s, as δ decreased from 16 µm to 12 µm, respectively, while Jeong et al. [51] reported from material analysis a grain refinement with reduction in d from 400–500nm to 50–90 nm with and without BHD realized by magnetic stirring, respectively. The grain refinement effect was purely attributed to the reduction in δ by BHD. Thereby, it shows the implications of the change in δ towards the electrochemical deposition of nC-materials.

### 5.2. Physical Effects of BHD on Nucleation and Growth Kinetics during ECD

**Figure 13:**
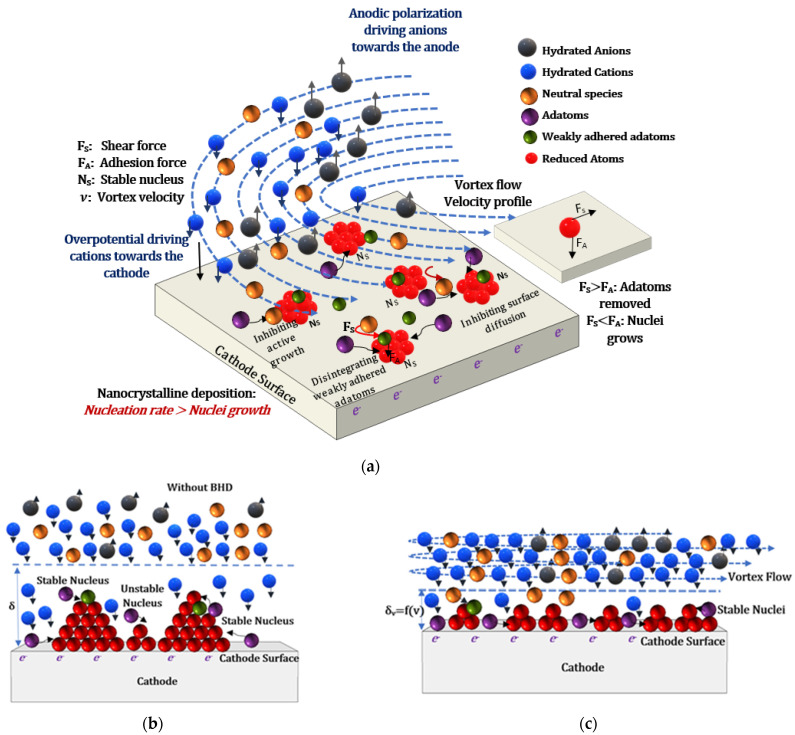
(**a**) Qualitative model showing nucleation kinetics with 3D nucleation and growth during ECD with BHD; (**b**) Schematic showing the implication of not using BHD on the diffusion layer thickness and the nucleation kinetics; and (**c**) Schematic showing the influence of BHD on the diffusion layer thickness and the respective nucleation kinetics.

A simple qualitative model is shown in Figure 13a–c for the physical effects of BHD on nucleation and growth kinetics during ECD where, similar to the preceding section, the 3D nucleation nature with diffusion-controlled growth of eNiCo alloy system is considered. The electroactive species, i.e., hydrated anions and cations, are represented by gray atoms and blue atoms, respectively. The neutral species in the electrolyte are shown as golden atoms. Owing to the potential gradient due to the polarization of electrodes, anions and cations are directed towards their respective electrodes, as shown by the small arrow heads in Figure 13a. The cations, after diffusing through the Nernst diffusion layer (shown by dashed line in Figure 13b,c), land on the cathode surface directed by the electrostatic forces (i.e., resulting from the polarization of the cathode), and eventually shed their hydration sheath and form the so-called adatoms (i.e., adsorbed atoms) shown as purple colored atoms in Figure 13a [54]. These adatoms diffuse across the cathode surface to the thermodynamically preferred sites such as edges, kinks, or already formed nuclei (shown as clusters of red adatoms in Figure 13a), where the phase transformation occurs by getting completely discharged and reduced as deposited metal atoms. Furthermore, this formation of stable clusters (radius > critical radius) of reduced atoms is referred to as nucleation, and further attachment of adatoms is referred to as grain growth. However, the clusters with radius < critical radius have a high probability of being dissolved again.

In the case of ECD without BHD, as discussed in the preceding section, without BHD, δ is large, which promotes grain growth (Figure 13b) and will result in growth of large nuclei and, furthermore, a kind of “shielding” effect occurs for the growth of nuclei in between the stable nuclei, thus resulting in coarse grained deposits. However, with BHD as shown by the dashed orbital lines, irrespective of the potential gradient, all the ions present in the electrolyte are also drifting across the cathode surface, and thus create a shear force when colliding with already formed nuclei. As described earlier, BHD reduces δ , as shown by the dashed line in Figure 13c and, hence, the distance from the surface at which a vertical movement of cations towards the electrode surface is observed reduces with BHD. This extra drift induced shear force will reduce the probability for the growth of large, columnar-like nuclei, and lead to a kind of soft polishing effect, as shown in Figure 13c. Additionally, the probability that weakly adhering nuclei are removed earlier from the surface is increased by BHD, thus reducing the size of critical nuclei which are stable. This will increase the chance that more smaller, thus denser, stable nuclei are formed, and will provide a further physical effect for the experimentally observed creation of less rough and more planar (fractal dimension changing from around 3 to 2) surfaces, as seen occurring in eNiCo and eNi coating samples shown in Figure 6 and Figure 7. This planarization effect can be attributed to the physical effects of BHD. As discussed in Section 4.3, $R_{a}$ values of eNiCo and eNi coating samples decreased by circa 90% and 80% as a function of increasing BHD up to v=42  cm/s, respectively. Additionally, the FD of these coating samples reduced from 2.97 to 2.15, and from 2.91 to 2.12 in the case of eNiCo and eNi coating samples, respectively. Additionally, Bigos et al. [43] recently reported a similar effect of planarization of surface morphology as a function of BHD in the case of nC-eNi coatings using BHD with a rotating disk electrode mode. There, $R_{a}$ values of the nc-eNi coatings decreased from 190 nm to 75 nm on increasing the BHD rotation rate from 100 rpm to 300 rpm. This phenomenon is explained in Figure 13a, where the green atoms representing weakly adhered adatoms are being detached from the cluster due to the increase in collision frequency $\omega_{c}$ with ions and neutral species as f(BHD) in the electrolyte.

Furthermore, it might also hinder the surface diffusion (i.e., by colloidal shear force) of the adatoms to the energetically favorable sites and stable nucleus, thereby reducing the attachment probability (frequency) $\omega_{a}$ and enhancing the detachment probability (frequency) $\omega_{d}$ . This results in inhibiting the growth of the stable nuclei, along with accelerating the ablation of unstable nuclei, thereby facilitating the cathodic reduction in new fresh nuclei, and hence increasing the nucleation density with finer grain size. This model can be linked to the classical nucleation theory, where the steady state nucleation rate $N_{o}\;$ is given by Equation (19) [55].

(19) $$N_{o}=N\cdot \left(\frac{\omega_{c}}{g_{c}}\right)\cdot {\left(\frac{-\Delta G_{c}}{3\pi k_{B}T}\right)}^{1/2}\cdot exp\left(\frac{-\Delta G_{c}}{k_{B}T}\right)$$

where N is the number of active sites on electrode, $\;g_{c}$ is the number of atoms in the nucleus, $\Delta G_{c}$ is the formation energy of the critical nucleus, and $\omega_{c}$ is considered as the frequency of collision of adatoms with the critical nucleus, which is increased by BHD; however, in respect to [55], $\omega_{c}$ is related to current and, therefore, to drift velocity $v_{e}$ in an electric field only. This relation shows the dependence of nucleation rate $N_{o}$ with $\omega_{c}$ , thereby indicating the physical effects of BHD on nucleation and growth kinetics. Furthermore, considering that, at a global scale, the moving electrolyte exerts shear forces $F_{s}$ across the cathode surface, and if unstable clusters of nuclei and the adatoms are adhered to the stable nuclei with a weak adhesion force $F_{A}$ , then in this case, i.e., $F_{s}>F_{A}$ , they get stripped off, thereby increasing $\omega_{d}$ . However, a very peculiar behavior of saturation in the degree of planarization of surface morphology and the grain-refinement effect for BHD > $v_{c}$ is observed, which is evident from computed d (Figure 10a) and from SEM and AFM micrographs (Figure 6 and Figure 7). This effect can be attributed, as shown in Figure 13a, to the interaction between the electrolytic fluidic shear and the growth kinetics, where at $v_{c}$ with reduced d , the shear forces acting on the stable nuclei are less than the adhesion forces of stable nuclei, thereby, a saturation in the influence of BHD on grain growth effect occurs, and hence no significant improvement of surface roughness occurs with increasing $v>v_{c}$ . A similar outcome can be seen in the work of Qiao et al. [27], where computed d decreased from 18.5 nm to 15 nm on increasing the BHD jet speed from 417 cm/s to 834 cm/s, which gives an indication of the saturation effect of BHD on the nucleation and growth kinetics.

Therefore, from the preceding results, it is evident that by keeping all ECD operating conditions constant and by exclusively varying BHD conditions, the nature of phase transformation occurring on the cathode surface (i.e., nucleation and growth kinetics) is significantly affected, which is manifested by change in the surface morphology and the grain size as shown by SEM, AFM micrographs, XRD diffractograms, and the resultant microhardness characterization (Section 4) of the eNiCo and eNi coatings samples. The changes in average surface roughness as a function of varying BHD conditions is found to be most sensitive with 94% and 81% reduction in $R_{a}$ values on increasing BHD up to v=42 cm/s for eNiCo and eNi coating samples, respectively, while the computed grain size reduced by 61% and 62% on increasing BHD up to v=42 cm/s for eNiCo and eNi, respectively. Consecutively, the microhardness values increased by 76% and 49% on increasing BHD up to v=42 cm/s for eNiCo and eNi, respectively. However, the interplay between BHD and other ECD operating conditions has to be investigated further. Furthermore, as described in Section 3, BHD can be implemented by several approaches, e.g., the direct flow of electrolyte towards the cathode surface, rotating disk electrode, sonication agitation mode, centrifugal mode, etc. The BHD method used in this paper can be grouped with those methods that would result in a similar laminar flow of electrolyte towards the cathode surface, unless a perforated membrane is held in front of the cathode, which would then significantly influence the flow regime towards the cathode surface. However, considering the ultrasonic mode of BHD and much higher electrolyte velocities used, for example, in [27], the BHD kinetics at the cathode would be different and, correspondingly, the effects of BHD on nucleation and growth kinetics would be different. In the range of electrolyte velocity by BHD investigated here (0–42 cm/s), both the electrochemical, as well as the physical effects, have an influence on the grain size, which can be deduced from the different dependence of diffusion layer thickness δ and grain size (Figure 12a,b) on electrolyte velocity v where d shows a larger slope at lower v values and a more pronounced saturation at larger v values than δ .

## 6. Conclusions

The influence of bath hydrodynamics realized by magnetic stirrer on the nucleation, growth kinetics and the resultant micromechanical properties of electrodeposited nickel-cobalt alloy system is investigated. The primary findings of this study can be summarized as follows:

1. The convective velocity profile within the vortex created by magnetic stirrer has an inhomogeneous nature across the electrolytic cell, wherein the electrolytic flow region with a minimum variation occurs near the boundary of the electrolytic cell;
2. The degree of anomaly *d_A_*, in the case of electrodeposited nickel-cobalt alloys, increased initially strongly as a function of increasing bath hydrodynamic velocity, i.e., from *d_A_* = 7.5 to *d_A_* = 15.7 for an increment in BHD velocity from 0 to 26 cm/s, and it only gradually increased from *d_A_* 15.7 to 17.2 for bath hydrodynamics beyond the critical velocity up to investigated maximum velocity of 42 cm/s. This finding is also a proof that the chosen BHD provides, by magnetic stirring, a suitable range of velocities to investigate the influence of BHD;
3. The surface morphology of electrodeposited nickel-cobalt alloy and nickel coating samples changed from granular to more planar as a function of increasing bath hydrodynamic velocity, indicating the electrodeposition of fine grained and compact coatings;
4. The AFM micrographs showed that the average surface roughness and the fractal dimension values decreased with increasing bath hydrodynamic velocity, i.e., it decreased from 207 nm (FD = 2.97) to 11 nm (FD = 2.15), and from 181 nm (FD = 2.91) to 33 nm (FD = 2.2) on increasing the velocity from 0 to 42 cm/s for nickel-cobalt and nickel coatings, respectively;
5. The X-ray diffraction characterization of electrodeposited nickel-cobalt alloys and nickel coating samples revealed, firstly, the fcc nature of the coatings, and secondly, showed a peak broadening of the diffractograms as a function of increasing bath hydrodynamic velocity;
6. The computed grain size using the Debye–Scherrer relation from the diffractograms decreased from 31 nm to 12 nm, and 69 nm to 26 nm as function of increasing bath hydrodynamic velocity (up to 42 cm/s) for nickel-cobalt and nickel coating samples, respectively;
7. Consecutively, the microhardness increased by 76% (i.e., from 393 HV0.01 to 692 HV0.01), and by 49% (from 255 HV0.01 to 381 HV0.01) on increasing the convective velocity from 0 to 42 cm/s for nickel-cobalt and nickel coating samples, respectively, which fits well with the Hall–Petch relationship.

The influence of bath hydrodynamics is modelled using two phenomena, namely, the electrochemical phenomenon, considering the implications of bath hydrodynamics on the resultant thickness of diffusion layer, and the physical phenomenon, considering the fluid-cathode interactions and the according shear forces acting on the adatoms. The computed diffusion layer thickness is found to decrease by 12% (for Ni) and 17% (for NiCo) on increasing the convective velocity from 0 to 42 cm/s, which is found to have a correlation with the decrement in the grain size as function of bath hydrodynamics. Therefore, the bath hydrodynamics exerts electrochemical and physical effects on nucleation and growth kinetics, thereby enhancing ablation of unstable nuclei and weakly adhered adatoms and, consecutively, inhibiting the grain growth. This subsequently results in a steady rate of nucleation and growth behavior, which consequently results in the grain-refinement effect and, hence, enhances the resultant micromechanical properties, i.e., microhardness and yield strength for the electrodeposited NiCo and Ni films.

## Figures and Tables

**Table 1:** Electrolyte composition and ECD operating conditions.

Electrolyte Constituents and ECD Operating Conditions	
Ni(NH_2_SO_3_)_2_.6H_2_O	1.8 (mol/L)
Co(NH_2_SO_3_)_2_.6H_2_O	0.03 (mol/L)
H_3_BO_3_	0.5 (mol/L)
Deionized H_2_O	14 (mol/L)
Bath pH value	4
Temperature	45 ± 2 °C
Current Density	50 mA/cm^2^

**Table 2:** Summary of electrochemical and micromechanical characterization of the eNiCo and eNi coating samples as function of BHD with varying v at j = 50 mA/cm^2^.

Sample	v (cm/s)	E_WE_ (V)	t (µm)	Co_A_ (wt%)	d_A_	R_a_ (nm)	FD	XRD FWHM (2θ°)	d (nm)	H (HV0.01)
eNiCo alloy coatings	0	−0.89 ± 0.016	13.5 ± 0.9	15.1	7.5	207.5 ± 9.8	2.97	0.29	31.83	393 ± 12
	8.8	−0.76 ± 0.004	13.6 ± 1.1	21.6	10.8	197 ± 7	2.89	0.37	24.95	462 ± 18
	18	−0.66 ± 0.005	12.4 ± 0.8	27.8	13.9	138.6 ± 8.5	2.77	0.66	13.98	570 ± 11.5
	26	−0.61 ± 0.013	12.8 ± 1.3	31.4	15.7	31.3 ± 6.7	2.65	0.68	13.57	600 ± 17.8
	34	−0.59 ± 0.010	12.6 ± 1.4	31.3	15.6	23.9 ± 3.9	2.21	0.66	13.98	613 ± 9.7
	42	−0.57 ± 0.006	12.8 ± 1.4	34.4	17.2	11.1 ± 1.65	2.15	0.76	12.14	692 ± 21
eNi coatings	0	−1.27 ± 0.016	44.8 ± 0.9	-	-	181.7 ± 8.7	2.91	0.13	68.95	255 ± 13.8
	12	−1.08 ± 0.02	42.2 ± 1.1	-	-	157.5 ± 7.7	2.68	0.18	50.21	287 ± 17
	22	−1.05 ± 0.02	42.7 ± 1.3	-	-	114 ± 6.9	2.54	0.28	31.42	315 ± 22
	32	−0.93 ± 0.016	41.4 ± 0.8	-	-	51 ± 7.4	2.4	0.29	31.19	325 ± 17.8
	42	−0.92 ± 0.01	42.6 ± 0.8	-	-	33.4 ± 1.9	2.2	0.34	26.19	381 ± 4.7

## Data Availability

Not applicable.
