# Correction: Khazi et al. Influence of Bath Hydrodynamics on the Micromechanical Properties of Electrodeposited Nickel-Cobalt Alloys. *Materials* 2021, *14*, 3898

**DOI:** 10.3390/ma17246234

**Published:** 2024-12-20

**Authors:** Isman Khazi, Ulrich Mescheder, Jürgen Wilde

**Affiliations:** 1 Institute for Microsystems Technology (iMST), Faculty of Mechanical & Medical Engineering, Robert Gerwig-Platz 1, 78120 Furtwangen im Schwarzwald, Germany; mes@hs-furtwangen.de; 2 Department of Microsystems Engineering (IMTEK), University of Freiburg, Georges-Köhler-Allee 103, 79110 Freiburg im Breisgau, Germany; juergen.wilde@imtek.uni-freiburg.de; 3 Associated to the Faculty of Engineering, University of Freiburg, 79110 Freiburg im Breisgau, Germany

There was an error in the original publication [1]. The relative percentage change values for the microhardness increase as function of bath hydrodynamics for nickel–cobalt alloys should be 76% instead of 43% and for nickel coatings should be 49% instead of 33%.

In the Abstract, 43% has been changed to 76% and 33% has been changed to 49%.

In Section 4.5., Microhardness Characterization of eNi and eNiCo Alloy Samples, paragraph 1, 33% has been changed to 49% and 44% has been changed to 76%.

In Section 5.2., Physical Effects of BHD on Nucleation and Growth Kinetics during ECD, paragraph 4, 43% has been changed to 76% and 33% has been changed to 49%.

In Section 6., Conclusions, paragraph 1, 43% has been changed to 76% and 33% has been changed to 49%.

Since the corresponding author’s original email address and telephone number are no longer in use, the telephone number has been updated to “+49-1590-4852-181”, and the email address has been updated to “isman.khazi@imtek.uni-freiburg.de”.

The authors state that the scientific conclusions are unaffected. This correction was approved by the Academic Editor. The original publication has also been updated.
